# Intestinal microbiota contributes to the heterogeneity of fat deposition by promoting mitochondrial fatty acid β-oxidation

**DOI:** 10.1080/19490976.2025.2593076

**Published:** 2025-12-03

**Authors:** Lukuan Li, Nannan Zhou, Zhe Wang, Tong Wang, Yuexin Wang, Fang Qiao, Zhen-Yu Du, Mei-Ling Zhang

**Affiliations:** a Laboratory of Aquaculture Nutrition and Environmental Health (LANEH), School of Life Sciences, East China Normal University, Shanghai, People's Republic of China; b Institute of Advanced Agricultural Science and Technology, East China Normal University, Shanghai, People's Republic of China

**Keywords:** C. somerae, β-oxidation, L-Carnitine, mesenteric fat

## Abstract

The gut microbiota plays a crucial role in lipid metabolism in both humans and animals. However, the specific contributions of gut microbiota and their associated metabolites to fat deposition, as well as the underlying mechanisms, remain largely unexplored. In this study, we demonstrated that the intestinal microbiota mediated the heterogeneity of mesenteric fat index (MFI), as evidenced by fecal microbiota transplantation (FMT) experiments. Notably, analysis of the 16S rRNA gene amplicon sequencing of 44 samples revealed a significantly higher abundance of *Cetobacterium somerae* in the Low MFI group, with a positive correlation to reduced MFI. Serum metabolomics analysis confirmed that L-Carnitine emerged as the most differentially abundant metabolite in the Low MFI group and exhibited a strong positive correlation with *C. somerae* abundance. Metagenomic analysis showed that microbial genes related to L-Carnitine biosynthesis were significantly enriched in the Low MFI group. Further, *C. somerae* was isolated and cultured, and its subsequent monocolonization in germ-free zebrafish and tilapia demonstrated its lipid-lowering effects by enhancing mitochondrial fatty acid *β*-oxidation. Whole genome sequencing demonstrated *C. somerae* could encode the [EC:1.2.1.3] gene, which promotes the production of 4-trimethylammoniobutanoate, a precursor of L-Carnitine, thereby enhancing L-Carnitine biosynthesis by the host and gut microbiota, leading to the reduced fat deposition in Nile tilapia. In conclusion, *C. somerae*, a core gut microbe with high abundance in aquatic teleost intestines, plays an important role in host lipid metabolism. This study advances our understanding of how core gut microbes shape host phenotypes and provides novel insights into manipulating core gut colonizers to reduce fat deposition.

## Graphical abstract

**Figure:**
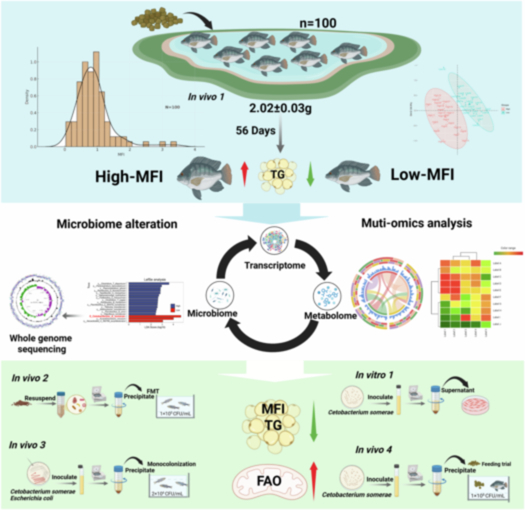

## Introduction

The gastrointestinal tract harbours a highly complex and dynamic microbial ecosystem that profoundly influences host physiology through its roles in nutrient metabolism, immune modulation, and maintenance of homoeostasis.^1‐3^ With the development of multi-omics technologies, the gut microbiota is increasingly recognised as a key regulator of systemic metabolic processes, acting through host–microbe interactions along the gut–organ axis.^4^^,^^5^ Among these, lipid metabolism appears especially sensitive to microbial modulation. However, despite growing insights into the microbiota’s role in host metabolism, the mechanisms by which gut microbes contribute to inter-individual variability in lipid metabolism remain poorly understood.

Mechanistic studies indicate that gut microbes can influence host lipid metabolism through both direct and indirect pathways. For example, the phylum Firmicutes, a dominant component of the gut microbiota, secretes abundant carbohydrate-active enzymes (CAZymes) to degrade dietary fibre and complex nutrients, thereby increasing host absorption of energy substrates.^6^^,^^7^ Consistent with this, Semova et al. demonstrated that Firmicutes mainly increase the number of lipid droplets in intestinal epithelial cells, while strains of the Bacteroidetes and Proteobacteria mainly enlarge the lipid droplets. Beyond these direct effects, microbe-derived metabolites—including bile acids,^8^ short-chain fatty acids,^9^ indole derivatives,^10^ trimethylamine *N*-oxide,^11^ fatty acids,^12^ and amino acids^13^—can reach target organs via the venous circulation and thereby influence host lipid metabolic responses.^5^

In recent years, studies in heterogeneity experiments have shown that the gut microbiota can mediate the heterogeneity of fat deposition. Wen et al. (2024) reared 40 mice under the same nutritional conditions for 12 weeks and observed heterogeneous obese phenotypes among the mice. They further identified that the gut microbe *Bacteroides vulgatus* mediated this obesity heterogeneity.^14^ Similarly, Ma et al. (2024) raised 78 Jinhua pigs in the same environment and under the same nutritional regime for 90 d, and also found heterogeneous obese phenotypes in the pigs. Subsequent experiments confirmed that *Clostridium butyricum* and its metabolite butyrate mediated the heterogeneity of these obese phenotypes.^15^ However, many of these studies have been limited by host heterogeneity in genetic background, which complicate interpretation of microbe–host causal relationships.

To overcome these limitations, we employed Nile tilapia (*Oreochromis niloticus*) as a model. Compared to mice and other livestocks, Nile tilapia offers several experimental advantages, including high fecundity and moderate body size making it well-suited for dissecting host–microbiota interactions. In this study, 100 male individuals with uniform genetic backgrounds and an initial body weight of 2.02 ± 0.03 g were reared under identical dietary and environmental conditions for 56 d. Despite similar body weights, the fish developed markedly different levels of mesenteric fat deposition at the end of the experimental period. Next, we integrated hepatic transcriptomics, serum metabolomics, intestinal metagenomics, and faecal microbiota transplantation to identify microbial determinants of adiposity divergence. This approach pinpointed *Cetobacterium somerae* as a key taxon associated with fat heterogeneity. Through functional characterisation, whole-genome sequencing, and targeted metabolite profiling, we delineated a previously unrecognised mechanism by which *C. somerae* modulates host adiposity phenotype, providing new insights into microbe-mediated regulation of fat storage in vertebrate models.

## Methods

### Animal trial 1: characterising the inter-individual variation in abdominal fat deposition in tilapia

Nile tilapia were obtained from the same parent stock in Guangzhou Tianfa Fry Development Co., Ltd. Prior to the feeding trial, the fish were acclimated to the culture water environment for two weeks. During the feeding trial, a total of 100 healthy tilapia (2.02 ± 0.03 g) with identical sex (male), genetic background and uniform body size were reared in a recirculating aquaculture system with a total volume of 1,400 L for eight weeks, ensuring that all experimental fish were maintained under identical water conditions. To ensure a consistent nutritional background, all tilapia were fed the same diet twice daily throughout the feeding trial. The feed formulation was provided in Table S1. The culture parameters were controlled as follows: water temperature 27−30 °C, ammonia-*N* < 0.2 mg/L, nitrite-*N* < 0.025 mg/L, dissolved oxygen > 6.5 mg/L, pH 7.3 ± 0.2, and a photoperiod of 14 hours light and 10 hours dark, with lighting from 8:00 am to 10:00 pm.

After the 8-week experimental period, all fish were euthanized using MS−222 (20 mg/L, Western Chemicals Inc., USA), and weighed individually. The anaesthetised fish was placed on a clean dissecting tray, and the abdominal cavity was then opened using dissecting scissors to separate the visceral mass. Liver and mesenteric fat tissues were dissected and weighed to calculate hepatosomatic index (HSI) and mesenteric fat index (MFI). Then, fish with the lowest MFI values were selected as the Low group, accounting for 22% of the total population. To enable comparison, fish with the highest MFI values were selected as the High group at the same proportion. We chose 44 MFI samples with extreme phenotype values for grouping. The 22 fish with the low MFI values (0.35 ± 0.04) were assigned to the Low mesenteric fat group (Low group), while the 22 fish with the high MFI values (1.34 ± 0.08) were assigned to the High mesenteric fat group (High group).

HSI(%)=(Liver weight/Body weight/)*100;

MFI(%)=(Mesenteric fat weight/Body weight)*100

### Animal trial 2: faecal microbiota transplantation in germ-free (GF) zebrafish

The experimental design was shown in Figure 4G. The production of germ-free zebrafish was described in supplementary materials. Faecal microbiota suspensions were prepared following the previously described methods.^16^ Briefly, the intestinal contents from six fish of the same group were collected and rinsed with sterile water for three times. The contents were then suspended in 5 mL of sterile water. The mixture was centrifuged three times at 1000 rpm for 1 minute to remove the supernatant, which was then centrifuged at 7500 rpm for 10 minutes to collect the precipitate. The bacterial suspension was subjected to gradient dilution and inoculated onto LB plates, which were incubated at 28 °C for 16 hours for bacterial growth. The intestinal bacterial cells of Nile tilapia from High or Low group were added to sterilised gnotobiotic zebrafish medium, containing 0.06 mg/mL marine salt (GZM) and at 3 d post-fertilisation (dpf). GF zebrafish larvae were transferred into a 10-cm petri dish using sterile pipettes, and the bacteria were added to achieve a final concentration of 1 × 10^6^ CFU/mL in GZM. GF zebrafish colonised with intestinal bacteria from the High and Low groups were designated as High-FMT and Low-FMT, respectively. Each group consisted of three replicates, with each replicate containing 50−60 alive zebrafish. Two groups were sampled 4 d post-faecal microbiota transplantation (7 dpf).

### Animal trial 3: *Cetobacterium somerae*monocolonization in Germ-free zebrafish

The experimental design is shown in Figure 6A. *C. somerae* was isolated from the intestinal contents of the experimental tilapia. *C. somerae* was cultured in sterile Gifu anaerobic medium (GAM) (Haibo, China) at 28 °C for 14 h in the anaerobic chamber. The bacterial culture was centrifuged at 7500 rpm for 10 minutes to discard the supernatant. The resulting cell pellet was washed three times with sterile water, then resuspended in sterile Gnotobiotic Zebrafish Medium (GZM) and inoculated into the GZM at a final concentration of 2 × 10^6^ CFU/mL. At 3 dpf, GF zebrafish larvae were transferred into 10 cm diameter petri dishes using sterile pipettes. The larvae were then aseptically divided into three treatment groups: GF-Control, GF mono-associated *C. somerae* (Ceto group), and GF mono-associated *Escherichia coli* (*E.coli* group). Each group included three replicates, with each replicate containing 50−60 live zebrafish. Inoculations were carried out every two days until day 14, and the three groups were sampled at 15 dpf.

### Animal trial 4: the administration of live*C. somerae* in tilapia

The experimental design is shown in Figure 7A. Juvenile Nile tilapia were also obtained from Guangzhou Tianfa Fry Development Co., Ltd. and underwent a 14-day acclimatisation period before the formal feeding trial. A total of 180 fish, with an average initial weight of 5.50 ± 0.01 g, were randomly assigned to two dietary groups: a control diet (Con), a control diet supplemented with *C. somerae* (Con + Ceto), and housed in 120-L tanks within individual tank. Each treatment group had three replicate tanks, with 30 fish per tank. The fish were fed a daily ration equivalent to 4% of their body weight, with biweekly adjustments based on their growth and the feed formulation detailed in Table S2. Sample collection methods were identical to those used in Animal Trial 1. Water quality was monitored and maintained consistently throughout the experiment, with parameters including water temperature 27−30 °C, ammonia-*N* < 0.2 mg/L, nitrite-*N* < 0.025 mg/L, dissolved oxygen >6.5 mg/L, pH 7.4 ± 0.2, and a photoperiod of 14 hours light and 10 hours dark, with lighting from 8:00 am to 10:00 pm. The culturing method for *C. somerae* was described earlier. After cultivation, the cells were harvested by centrifugation, resuspended in sterile water, and incorporated into the diet, achieving a final concentration of 1 × 10^8^ CFU/g. Fresh batches of the diet were prepared daily and stored at 4 °C.

### Cell culture and treatment

Primary hepatocytes were isolated from healthy Nile tilapia as previously described.^17^ In brief, liver tissue was rinsed three times with 1 × PBS and subjected to digestion using Dulbecco's Modified Eagle's Medium (DMEM) supplemented with 0.1% collagenase IV (17104019, Gibco, Suwanee, GA, USA), then filtered through a 70 µm nylon mesh. Hepatocytes were collected by centrifugation at 1050 × g for 10 minutes. Red blood cells were removed using a red blood cell lysis buffer (RT122−02, Tiangen, Beijing, China), and the remaining cells were cultured in DMEM supplemented with 10% FBS and 1% penicillin-streptomycin at 28 °C in a cell culture incubator.

To investigate whether the supernatant of *C. somerae* could regulate lipid metabolism in tilapia, primary hepatocytes were treated with 250 µM sodium oleate + GAM (PYG), and 250 µM sodium oleate combined with culture supernatants from *C. somerae* at concentrations of 1 × 10⁸ CFU/mL (CS), 2 × 10⁸ CFU/mL (2 × CS), and 4 × 10⁸ CFU/mL (4 × CS) respectively, for 24 hours.

Triglyceride (TG) levels were quantified using a commercial kit (E1013, Applygen, China), and lipid droplets in tilapia hepatocytes were stained with a fluorescent staining kit (C2053S, Beyotime Biotechnology, China) following the manufacturer’s instructions. Cells were cultured in 24-well plates, collected, and washed three times with PBS before incubation with 0.1% BODIPY 493/503 and 0.1% Hoechst 33342 solution in the dark at room temperature for 30 minutes. After two washes with PBS, cell images were captured using an inverted fluorescence microscope (Revolve FL, USA), and BODIPY 493/503-positive areas were quantified using ImageJ software (NIH, Bethesda, USA).

### Histological observation

For histological observation, tissues were fixed in 4% paraformaldehyde for 24 hours. Then, the liver and mesenteric fat samples were dehydrated and embedded in paraffin, and sectioned at a thickness of 5 μm. The sections were stained with hematoxylin-eosin (H&E), followed by rinsing with 70% alcohol. For Oil Red O staining, liver tissue was embedded in optimal cutting temperature (OCT) compound (Sakura) and immediately frozen at −80 °C. Sections, approximately 8 μm thick, were briefly washed with 60% isopropanol. The frozen liver sections were then stained with Oil Red O and counterstained with hematoxylin to visualise lipid droplets.

For Nile Red staining, the fixed zebrafish tissue was embedded in OCT and stored at −80 °C. The frozen tissue sections are re-warmed at room temperature, and moisture is controlled. Then, diluted Nile Red is applied to the marked tissue and incubated for 10 minutes. For DAPI staining, slides are washed in PBS (pH 7.4) using a decolorising shaker (3 times, 5 minutes each) before adding DAPI and incubating for 10 minutes in the dark. The slides are then sealed after another PBS washing step. Finally, fluorescence imaging is performed with specific excitation and emission wavelengths for DAPI and Nile Red. All the slides were then examined under a Echo Revolve microscope (RVL−100-M|| ||RVL−100-M, USA).

### Biochemical analysis

Levels of TG (Jiancheng, A110−1−1), total-cholesterol (T-CHO) (Solarbio, BC1985), and non-esterified fatty acids (NEFA) (Jiancheng, A042−2−1), malondialdehyde (MDA) (Jiancheng, A003−1−2), and aspartate aminotransferase (AST) (Jiancheng, C010−2−1) were quantified using biochemical assay kits, following the manufacturer's instructions.

### Quantitative real-time PCR and western blotting analysis

Total RNA was extracted from tilapia liver tissues or whole-body samples of zebrafish larvae using a commercial kit (Cat. No. RM201−02) and an automatic instrument (VNP-32P, Vazyme, China). RNA quality and quantity were assessed using a Nanodrop 2000 Spectrophotometer (Thermo, Waltham, USA). cDNA synthesis was performed with a commercial kit (Cat. No. 11121, Yeasen, China), and qRT-PCR was carried out using ChamQ SYBR qPCR Master Mix (Cat. No. Q311, Vazyme, China) according to the manufacturer's protocol. Primer sequences are provided in Table S3−4, and amplification efficiency was confirmed for all primer pairs. Relative mRNA expression was calculated using the 2^−ΔΔCT^ method.

For protein analysis, liver proteins were extracted using RIPA lysis buffer, and approximately 30−50 μg of protein was loaded onto SDS-PAGE gels (New Cell & Molecular Biotech Co., Ltd., P2013), transferred to a nitrocellulose membrane, and blocked with 5% BSA. The membranes were then subjected to immunoblotting with primary and secondary antibodies, including anti-CPT1A (Proteintech, China), anti-ACAA2 (Abconal, China), anti-*β*-actin (Proteintech, China), anti-*α*-tubulin (Abconal, China), anti-GAPDH (bioworld, China) and anti-rabbit IgG (LI-COR, USA).

### Transcriptome analysis of liver

Total RNA was extracted from liver tissue samples, with seven biological replicates per treatment group. RNA quality was assessed, and the purified RNA was used for library preparation with the Illumina TruSeq™ RNA Sample Prep Kit (Illumina, San Diego, CA, USA). Paired-end sequencing was performed on the Illumina NovaSeq 6000 platform by Shanghai Personalbio Technology Co., Ltd. (Shanghai, China).

## 16S rRNA amplicon sequencing

The contents of the entire intestine of 22 samples from each group were collected and the gut microbial composition was analysed using 16S rRNA amplicon sequencing on the Illumina MiSeq PE300 platform, targeting the V3-V4 regions of the bacterial 16S rRNA gene. Amplicon sequence variants (ASVs) were generated through sequence dereplication using the divisive amplicon denoising algorithm 2 (DADA2). Sequencing data analysis was performed using the online platform at https://www.genescloud.cn Detailed methods for sequence analysis are available in a previous publication.^18^

### Shotgun metagenomic sequencing and analysis

Total microbial genomic DNA from the entire intestinal contents was extracted and sequenced at Personal Biotechnology Co., Ltd. (Shanghai, China). Raw reads were quality filtered using fastp (v0.23.2) to remove adaptor sequences and low-quality bases. High-quality reads were assembled using MEGAHIT, and contigs ≥300 bp were retained. Open reading frames (ORFs) were predicted using Prodigal, and non-redundant gene catalogues were generated using MMseqs2 clustering. Gene abundance was quantified by aligning quality-filtered reads to the non-redundant catalogue using featureCounts. Functional annotation was performed using MMseqs2 (search mode, -s 5.7) against KEGG database.

### Whole-genome sequencing of *C. somerae*

The complete genomic DNA of *C. somerae* was purified, and the sequencing libraries were constructed for both Illumina and PacBio platforms. The sequencing was performed by Personalbio (Shanghai, China) with 100 × coverage. Raw sequencing data underwent quality trimming using Fastp, followed by genome assembly with Unicycler. The assembled genome of *C. somerae* was subsequently categorised and functionally annotated using the eggNOG and KEGG databases.

### Untargeted metabolite analysis

The intestinal contents of 10 individuals from each group were collected for untargeted metabolite analysis using liquid chromatography-mass spectrometry (LC-MS). The metabolomics procedures, including LC settings (Thermo Vanquish with an ACQUITY UPLC HSS T3 1.8 μm, 2.1 × 150 mm column), MS settings (Thermo Q Exactive HF-X), data preprocessing, and statistical analysis, were described in a previous report.^9^

### Targeted metabolite analysis

The GAM and *C. somerae* fermentation supernatant (CetoST) were thawed on ice, and 200 μl of each was added to a pre-chilled methanol/acetonitrile/water solution (2:2:1 ratio by volume) and mixed. The sample was then sonicated at a low temperature for 30 minutes and frozen at 20 °C for 10 min, following which the samples were centrifuged for 20 minutes (14,000 g, 4 °C), and the collected supernatant was dried under vacuum. For LC–MS analysis, the samples were redissolved in 100 pL of aqueous solution (acetonitrile: water = 1:1 ratio by volume) with vigorous mixing, followed by centrifugation for 15 minutes (14,000 g, 4 °C) and the supernatant was collected. Analyses were performed using a UHPLC (1,290 Infinity LC, Agilent Technologies) coupled to a QTRAP MS (AB 6500+, AB Sciex) at Shanghai Personalbio Technology Co., Ltd (Shanghai, China). The metabolites were separated on HILIC (Waters UPLC BEH Amide column, 2.1 mm × 100 mm, 1.7 μm) and C18 columns (Waters UPLC BEH C18−2.1 × 100 mm, 1.7 μm). Finally, the measured metabolites were quantified using Multi Quant or Analyst software.

### Statistical analysis

The data for the measured parameters between the two dietary treatments were analysed for statistical significance using an independent t-test (SPSS version 25.0). One-way analysis of variance (one-way ANOVA) followed by Tukey’s post-hoc test to determine the statistical significance of differences between each pair of groups. A *P*-value of less than 0.05 was considered statistically significant. All quantitative results are expressed as mean ± standard error of the mean (S.E.M.). Chart analyses were performed using a free online data analysis platform (https://www.genescloud.cn;https://cnsknowall.com/). Co-occurrence analysis was visualised using Cytoscape v3.8.2 (http://www.cytoscape.org). Bar chart visualisation was conducted using GraphPad Prism 9.5.1. Figures were created using BioRender (https://www.biorender.com/).

## Results

### Nile tilapia with high and low MFI display distinct fat deposition patterns

**Figure 1.**
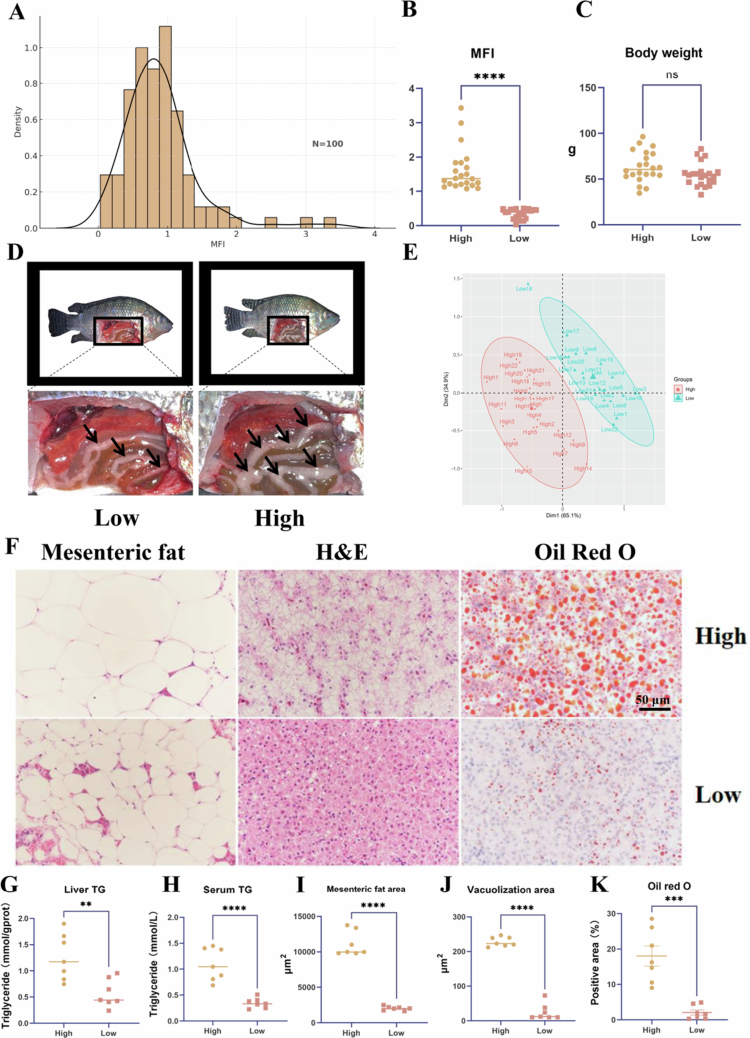
High and low MFI Nile tilapia exhibit distinct lipid deposition phenotypes. (A) Distribution of MFI in 100 Nile tilapia at the end of the feeding trial, showing a normal distribution. (B) Statistical comparison of MFI between the High and Low groups. (C) Body weight of tilapia in the High and Low groups. (D) Schematic representation of abdominal anatomy in High and Low MFI phenotypes. (E) Principal component analysis (PCA) plot showing separation between High and Low groups. (F) Representative images of mesenteric fat and liver tissue stained with hematoxylin and eosin (H&E, 400 × ), and liver sections stained with Oil Red O (Scale bar = 50 μm). (G) Hepatic triglyceride content in High and Low groups. (H) Serum triglyceride content in High and Low groups. (I) Quantification of mesenteric fat area in High and Low groups. (J) Quantification of hepatic vacuolisation area in High and Low groups. (K) Oil Red O-positive area in liver sections in High and Low groups. Data are presented as mean ± SEM. *P < 0.05 (Student's t-test). MFI: = 100* (Mesenteric fat/Body weight); High group: tilapia with high MFI; Low group: tilapia with low MFI; SEM: standard error of the mean.

To identify two cohorts with the greatest difference in MFI values, 100 tilapia were fed for 56 d, after which their MFI values were measured and ranked from lowest to highest. The distribution of MFI values followed a normal distribution (Figure 1A). Based on this distribution, two extreme cohorts were selected—representing the highest and lowest MFI values (*n* = 22 each; Figure 1B). No significant difference in body weight was observed between these two groups (Figure 1C), suggesting that the divergence in MFI was primarily attributable to differences in mesenteric fat deposition. Figure 1D visually depicts the contrast in mesenteric fat between the two cohorts, and Figure 1E further confirms that the difference in MFI was significant. To validate the phenotypic divergence, histological analysis of the liver and mesenteric fat was performed. The results revealed significantly lower lipid accumulation in the Low group compared to the High group (*P* < 0.001; Figure 1F, I–K). Additionally, liver TG, serum TG, liver HSI, serum AST, liver T-CHO, liver MDA, and liver NEFA levels supported the observed phenotypic separation and confirmed the reliability of cohort selection (Figure 1G–H; Figure S1).

### Transcriptomic heterogeneity in the liver of tilapia with high and low MFI groups

**Figure 2.**
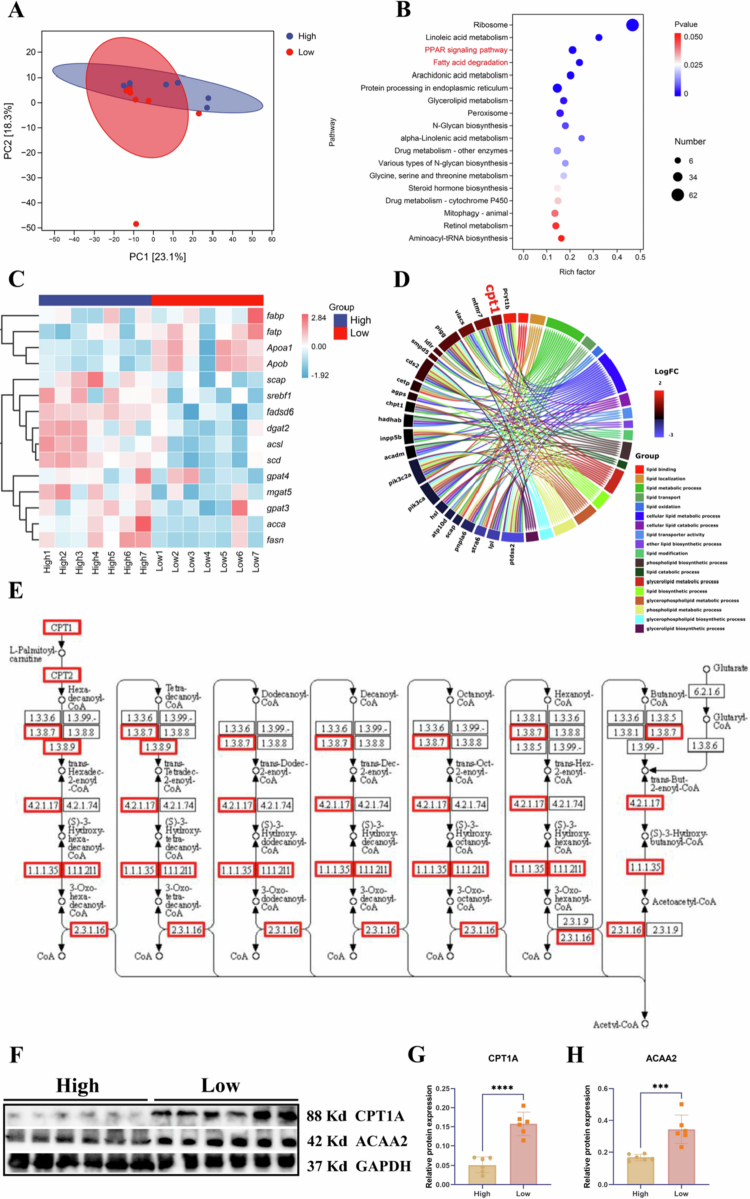
Liver Transcriptomic Heterogeneity Between Tilapia with High and Low MFI. (A) PCA plot showing transcript-level separation between the High and Low groups. (B) Top 18 differentially enriched KEGG pathways. (C) Heatmap of differentially expressed genes involved in lipid metabolism. (D) Enrichment analysis and chord diagrams of the top 18 lipid-related Gene Ontology (GO) terms. (E) Fatty acid degradation pathway highlighting significantly upregulated genes (red boxes) in the Low group. (F) Protein expression levels of CPT1A and EC 2.3.1.16 (ACAA2) in the High and Low groups. (G) Statistical analysis of CPT1A protein expression. (H) Statistical analysis of ACAA2 protein expression. Data are presented as mean ± SEM. *P < 0.05 (Student’s t-test). High group: tilapia with a high MFI; Low group: tilapia with a low MFI; SEM: standard error of the mean.

To investigate transcriptomic heterogeneity between the High and Low groups, the liver—a central organ of metabolic regulation—was selected for transcriptome analysis. As illustrated in Figure 2A, the two groups exhibited distinct hepatic gene expression profiles. KEGG pathway enrichment analysis identified significant enrichment of pathways associated with lipid metabolism (Figure 2B). Accordingly, genes involved in lipid synthesis, fatty acid biosynthesis, and lipid transport were further examined (Figure 2C). The results revealed that genes related to lipid and fatty acid synthesis were downregulated, whereas lipid transport genes were upregulated in the Low group. To further elucidate lipid-associated regulatory mechanisms, Gene Ontology (GO) enrichment analysis was performed, focusing on lipid metabolism-related terms (Figure 2D). Among the differentially expressed genes, *cpt1* was identified as a key candidate potentially contributing to the observed heterogeneity in fat deposition. Given that *cpt1* encodes the rate-limiting enzyme for mitochondrial *β*-oxidation, we subsequently analysed the expression of other genes involved in this pathway. The findings demonstrated that enzymes responsible for both the initiation and termination of mitochondrial fatty acid *β*-oxidation were significantly upregulated in the Low group (*P* < 0.05; Figure 2E). The expression patterns of these mitochondrial *β*-oxidation genes were further validated via qPCR in liver and mesenteric fat tissues (Figure S2 and Figure S3). These trends was also supported at the protein level by increased protein expression of CPT1A and EC:2.3.1.16 (ACAA2), the latter of which catalysers the final step of mitochondrial fatty acid *β*-oxidation (*P* < 0.001; Figure 2F–H).

### Non-target metabolomic heterogeneity in the serum of tilapia with high and low MFI groups

**Figure 3.**
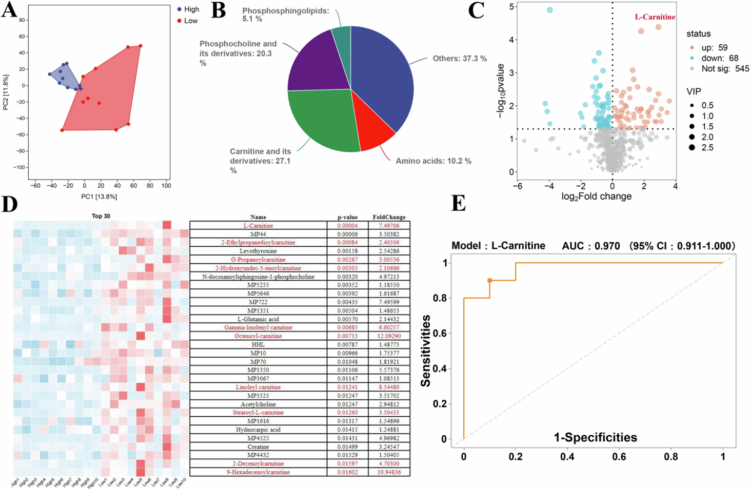
Metabolomic Heterogeneity in the Serum of Tilapia with High and Low MFI. (A) PCA plot analysis showing distinct separation of serum metabolites between the High and Low groups. (B) Pie chart of differentially expressed cationic metabolites. (C) Volcano plot displaying significantly altered metabolites between the High and Low groups. (D) Top 30 differentially cationic metabolites ranked by *P* value. (E) Receiver operating characteristic (ROC) curve analysis of L-Carnitine. Data are presented as mean ± SEM. *P < 0.05 (Student’s t-test). High group: tilapia with a high MFI; Low group: tilapia with a low MFI; SEM: standard error of the mean.

To investigate the metabolomic heterogeneity between the High and Low groups, serum metabolomic was conducted. PCA analysis revealed a clear separation between the two groups, indicating substantial differences in their serum metabolite compositions (Figure 3A). Figure 3B shows that 27.1% percent of the differential metabolites were significantly enriched in L-Carnitine and its derivatives. Among these, L-Carnitine emerged as the most prominent differential metabolite, exhibiting the lowest *P*-value and high abundance (Figure 3C and D; Table S5). Furthermore, Receiver Operating Characteristic (ROC) curve analysis identified L-Carnitine as having the highest discriminatory power, with an Area Under the Curve (AUC) value of 0.97, surpassing other carnitine-related metabolites (Figure 3E and Table S6).

### Gut microbiota mediated the heterogeneity of fat deposition

**Figure 4.**
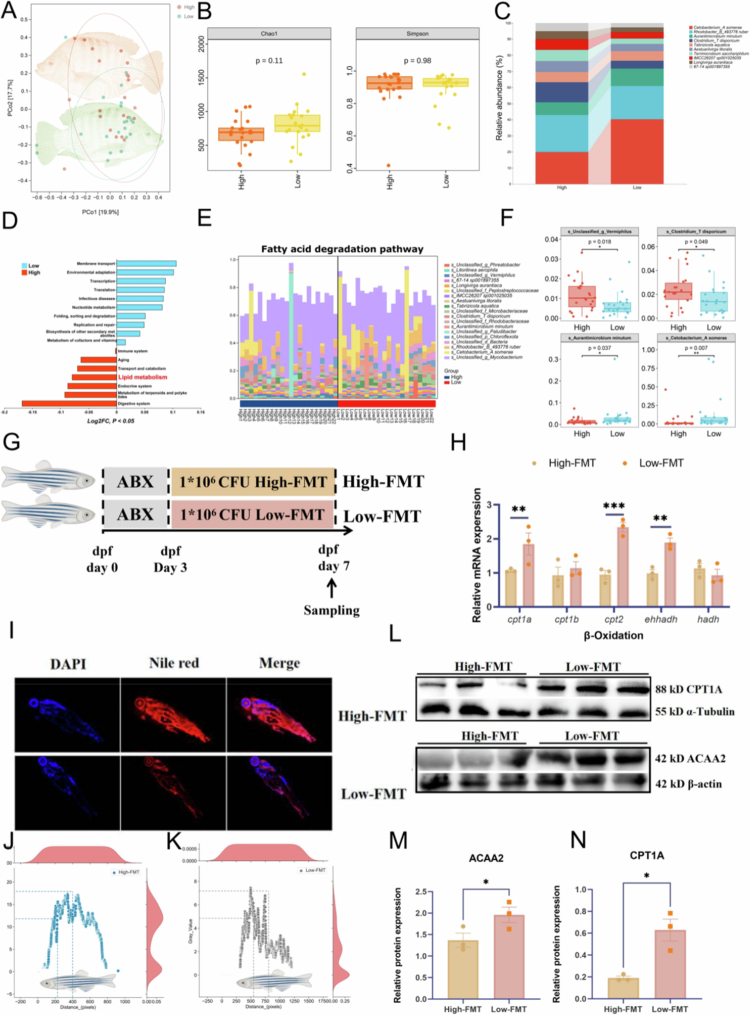
Gut microbiota contributed to MFI Heterogeneity. (A) Principal coordinates analysis (PCoA) showing *β*-diversity of intestinal microbiota between the High and Low groups. (B) Box plot of *α*-diversity indices comparing the two groups. (C) Taxonomic composition of intestinal microbiota at the species level. (D) Differentially enriched microbial metabolic pathways based on KEGG annotation (Log_2_FC, *P* < 0.05). (E) Top 20 microbial contributors to the fatty acid degradation pathway. (F) Four of the top 20 microbial contributors to the fatty acid degradation pathway were identified as significantly different between high and low groups. (G) Schematic diagram of the faecal microbiota transplantation (FMT) experiment. (H) Expression levels of genes involved in mitochondrial fatty acid *β*-oxidation in High-FMT and Low-FMT groups. (I) Nile red staining of zebrafish from High-FMT and Low-FMT groups. (J) Quantitative analysis of fluorescence intensity in High-FMT (K) Quantitative analysis of fluorescence intensity in Low-FMT. (L) Protein expression levels of CPT1A and ACAA2 in the High-FMT and Low-FMT groups. (M) Statistical analysis of ACAA2 protein expression. (N) Statistical analysis of CPT1A protein expression. Data are presented as mean ± SEM. *P < 0.05 (Student’s t-test). High group: tilapia with a high MFI; Low group: tilapia with a low MFI; SEM: standard error of the mean; High-FMT: zebrafish received faecal microbiota from high MFI donors.; Low group: zebrafish received faecal microbiota from low MFI donors.; SEM: standard error of the mean.

To elucidate the heterogeneity of fat deposition from a microbial perspective, 16S rRNA amplicon gene sequencing was performed on faecal samples. Microbial diversity analysis revealed no significant differences in *α*-diversity or *β*-diversity between the High and Low groups (Figure 4A and B). However, compositional analysis at the species level showed marked differences in the top 10 most abundant microbial taxa between the two groups (Figure 4C). These compositional shifts were accompanied by changes in microbial metabolic pathways, particularly those related to lipid metabolism (Figure 4D). Further analysis of the top 20 microbial contributors to the fatty acid degradation pathway revealed that *Vermiphilus* and *Clostridium disporicum* were downregulated in the Low group (*P* < 0.05), while *Aurantimicrobium minutum* and *Cetobacterium somerae* were significantly upregulated (*P* < 0.05) (Figure 4E and F).

To confirm whether the observed fat phenotype was mediated by the intestinal microbiota, FMT was performed. As illustrated in Figure 4G, faecal samples from the High and Low groups were transplanted into germ-free zebrafish, which were designated as High-FMT and Low-FMT, respectively. The expression levels of genes involved in mitochondrial *β*-oxidation, including *cpt1*, *cpt2*, and *ehhadh*, were significantly upregulated in the Low-FMT group (*P* < 0.05, Figure 4H). Nile red staining revealed markedly reduced lipid accumulation in the Low-FMT group compared to the High-FMT group (Figures 4I–K), as evidenced by significantly lower fluorescence intensity in the abdominal region. To further assess whether the intestinal microbiota influenced protein expression related to host mitochondrial *β*-oxidation, the levels of CPT1A and ACAA2, representing the rate-limiting enzymes at the initial and terminal steps of the pathway, were examined. As shown in Figure 4L–N, both CPT1A and ACAA2 protein levels were significantly elevated in the Low-FMT group compared to the High-FMT group (*P* < 0.05).

### *C. somerae* was indentified as a key species mediating fat deposition heterogeneity

**Figure 5.**
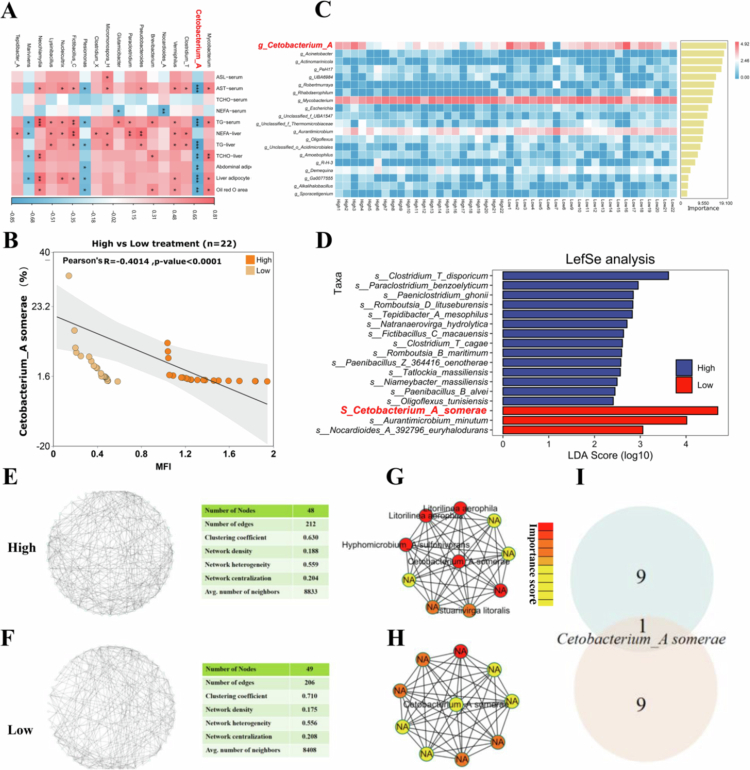
Identification of *C. somerae* as a Keystone Species Influencing MFI Heterogeneity. (A) Spearman’s correlation analysis between gut microbiota and physiological phenotypes (R > 0.6, *P* < 0.05). (B) Pearson’s correlation between *C. somerae* abundance and MFI. (C) Top 20 genera ranked by importance in random forest analysis. (D) LEfSe analysis identifying differentially abundant species (LDA score > 2). (E–F) Co-occurrence network analysis of top 50 abundant gut microbiota in the High and Low groups, respectively. (G–H) Identification of core microbial hubs in the High and Low groups, respectively, using the CytoHubba plugin in Cytoscape with the following settings: Node Scores-Calculate; Hubba Nodes-Top 10 ranked by degree; Display Options-Show shortest paths and expand subnetworks. (I) Identification of the shared bacterium in the core microbial hubs of the Low and the High groups is *Cetobacterium somerae*.

To identify the key microbial species involved in mediating fat heterogeneity, differences in gut microbiota composition between the High and Low groups were analysed. Spearman’s correlation analysis (Figure 5A) revealed that *C. somerae* was the most significantly species negatively correlated with TG, T-CHO, serum AST, and quantitative indicators of hepatic fat deposition (*P* < 0.01). Further analysis demonstrated a strong negative correlation between *C. somerae* abundance and MFI (*P* < 0.0001; Figure 5B). In addition, both random forest and LEfSe analyses consistently identified *C. somerae* as the most discriminative microbial feature distinguishing the two groups (Figure 5C and D). To further confirm core microbiota in mediating the fat deposition heterogeneity, co-occurrence network analyses of the gut microbiota were conducted. The global network topologies revealed similar structures between the two groups, with comparable numbers of microbial nodes and correlation edges (Figure 5E and F). To identify core microbial hubs, the CytoHubba plugin in Cytoscape was employed. Notably, although the High and Low groups exhibited distinct core subnetworks, both shared *C. somerae* as a central hub node (Figure 5G–I). Collectively, these findings suggest that *C. somerae* may play a critical role in modulating host metabolic phenotype and fat heterogeneity.

### Live *C. somerae* and Its metabolites regulate lipid metabolism via mitochondrial fatty acid *β*-Oxidation

**FIgure 6.**
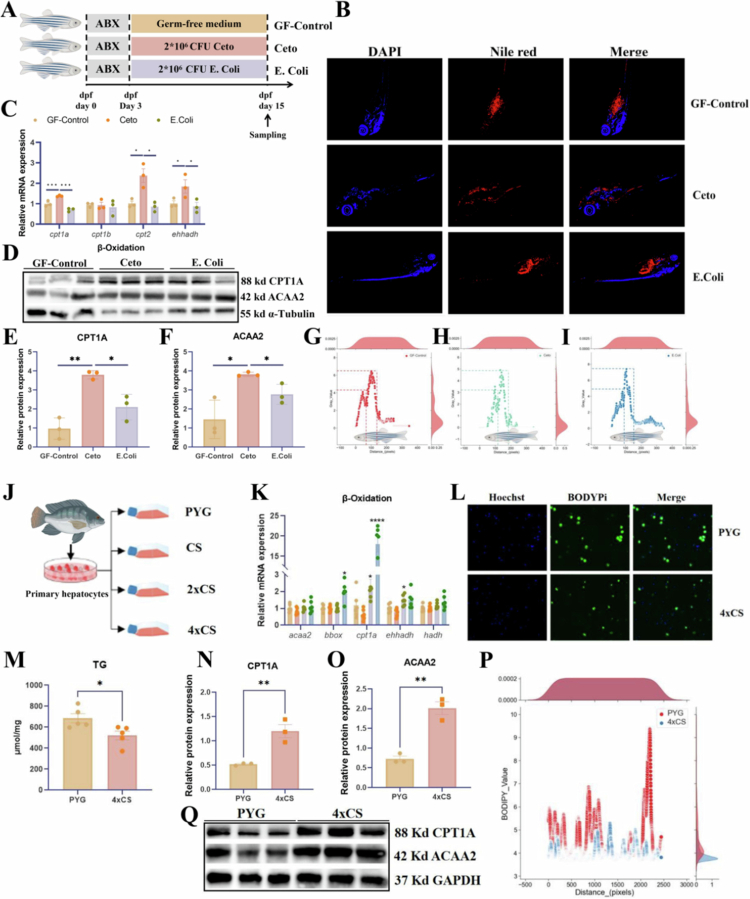
Live *C. somerae* and Its Metabolites Promote Mitochondrial Fatty Acid *β*-Oxidation to Regulate Lipid Metabolism. (A) Schematic diagram of the mono-colonisation experiments. (B) Nile red staining of zebrafish from GF-Control, Ceto, and *E. coli* group. (C) Expression levels of genes involved in mitochondrial fatty acid *β*-oxidation in GF-Control, Ceto, and *E. Coli* group. (D) Protein expression levels of CPT1A and ACAA2 in the GF-Control, Ceto, and *E. Coli* group. (E) Statistical analysis of CPT1A protein expression. (F) Statistical analysis of ACAA2 protein expression. (G) Quantitative analysis of fluorescence intensity in GF-Control (H) Quantitative analysis of fluorescence intensity in Ceto. (I) Quantitative analysis of fluorescence intensity in *E. coli*. (J) Schematic illustration of *C. somerae* supernatant treatment in primary tilapia hepatocytes. (K) Expression levels of genes related to mitochondrial fatty acid *β*-oxidation in PYG and *C. somerae* culture supernatant-treated groups. (L) BODIPY staining of primary tilapia hepatocytes in the PYG and 4 × CS groups. (M) TG content comparison between the PYG and 4 × CS groups. (*N*) Quantification of CPT1A protein expression levels. (O) Quantification of ACAA2 protein expression levels. (*P*) Fluorescence intensity analysis between the PYG and 4 × CS groups. (Q) Protein expression levels of CPT1A and ACAA2 in the PYG and 4 × CS groups. Data are presented as mean ± SEM. *P < 0.05 (Student’s t-test or one-way ANOVA). GF-Control: Germ-free zebrafish; Ceto: zebrafish colonised with live *C. somerae*; *E. coli*: zebrafish colonised with live *Escherichia coli*; PYG: Gifu anaerobic medium; CS, 2 × CS, and 4 × CS are culture supernatants from *C. somerae* at concentrations of 1 × 10⁸ CFU/mL, 2 × 10⁸ CFU/mL, and 4 × 10⁸ CFU/mL, respectively; SEM: standard error of the mean.

To investigate whether *C. somerae* specifically regulates host lipid metabolism, mono-colonisation experiments were performed using live *C. somerae* and *Escherichia coli* (Figure 6A). The results showed that Nile red staining revealed significantly reduced lipid accumulation in the Ceto group compared to both the GF-control and *E.coli* groups (Figure 6B), as evidenced by markedly lower fluorescence intensity in the abdominal region (Figures 6G–I). Furthermore, the expression levels of genes of *cpt1*, *cpt2*, and *ehhadh* were highest in the Ceto group (*P* < 0.05; Figure 6C). To further determine whether *C. somerae* influences protein expression associated with mitochondrial fatty acid *β*-oxidation, the levels of CPT1A and ACAA2 were examined (Figure 6D). As shown in Figures 6E and F, the protein levels of both CPT1A and ACAA2 were significantly elevated in the Ceto group compared to the GF-control and *E. coli* groups (*P* < 0.05).

To determine whether the lipid-lowering effects were attributable to the metabolites of *C. somerae*, the culture supernatant of *C. somerae* was incubated with primary hepatocytes isolated from tilapia (Figure 6J). The results showed that mRNA expression levels of *bbox*, *cpt1a*, and *ehhadh* were significantly upregulated in the 4 × CS group than in the PYG group (Figure 6K). Furthermore, measurements of ^14^C-palmitic acid *β*-oxidation efficiency in tilapia primary liver cells showed that this efficiency was significantly higher in the 4 × CS group than in the PYG group (Figure S4). BODIPY staining revealed a marked reduction in green fluorescence intensity in the 4 × CS group compared to the PYG control group, indicating reduced intracellular lipid accumulation (Figure 6L and P). Consistent with these findings, the TG content was significantly lower in the 4 × CS group than in the PYG group. Furthermore, Western blot further confirmed the lipid-lowering effect of the CS supernatant. The protein levels of CPT1A and ACAA2 were significantly increased in the 4 × CS group (*P* < 0.05, Figure 6N, O, and Q). This suggests that CS reduced lipid accumulation by enhancing mitochondrial fatty acid *β*-oxidation.

### Live *C. somerae* modulated MFI in tilapia via activating mitochondrial fatty acid *β*-oxidation in tilapia.

**Figure 7.**
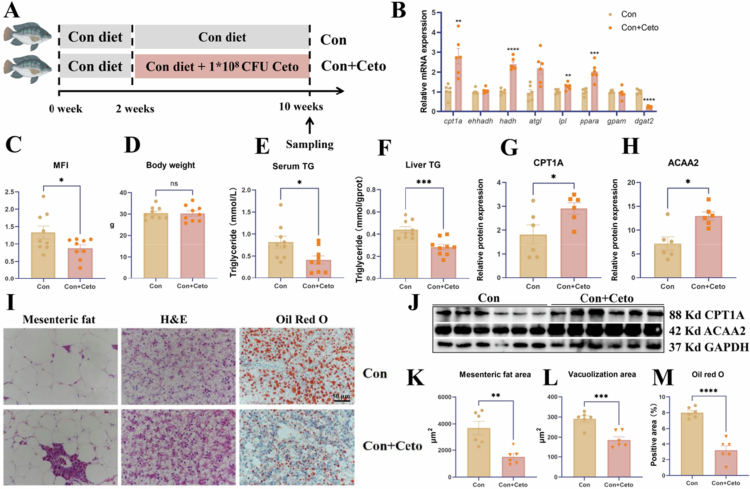
Administration of live *C. somerae* altered mesenteric fat deposition in tilapia through the activation of mitochondrial fatty acid *β*-oxidation. (A) Schematic diagram of the feeding trial experiment. (B) The expression levels of lipid-related genes between the Con and Con + Ceto. (C) Statistical comparison of MFI between the Con and Con + Ceto. (D) Body weight of tilapia in the Con and Con + Ceto groups. (E) Serum triglyceride content in the Con and Con + Ceto groups. (F) Hepatic triglyceride content in the Con and Con + Ceto groups. (G) Statistical analysis of CPT1A protein expression. (H) Statistical analysis of ACAA2 protein expression. (I) Representative images of mesenteric fat and liver tissue stained with hematoxylin and eosin (H&E, 400 × ), and liver sections stained with Oil Red O (Scale bar = 50 μm). (J) Protein expression levels of CPT1A and ACAA2 in the Con and Con + Ceto groups. (K) Quantification of mesenteric fat area in the Con and Con + Ceto groups. (L) Quantification of hepatic vacuolisation area in the Con and Con + Ceto groups. (M) Oil Red O-positive area in liver sections in the Con and Con + Ceto groups. Data are presented as mean ± SEM. *P < 0.05 (Student's t-test). Con group: tilapia fed with Con diet; Con + Ceto group: tilapia fed with live *C. somerae* diet; SEM: standard error of the mean.

To determine whether *C. somerae* could regulate MFI in tilapia, an 8-week feeding trial was conducted (Figure 7A). As shown in Figure 7B, the expression levels of lipolysis-related genes, including *cpt1a*, *hadh*, *lpl*, and *ppara*, were significantly upregulated in the Con + Ceto group compared to the control group (*P* < 0.01). In contrast, the expression of lipid synthesis genes was significantly downregulated in the Con + Ceto group (*P* < 0.0001). In addition, compared with the Con group, the expression levels of mitochondrial *β*-oxidation genes in mesenteric fat tissues were significantly elevated in the Con + Ceto group (Figure S5). Importantly, although there was no significant difference in body weight, the MFI was significantly reduced in the Con + Ceto group compared to the Con (Figure 7C and D). Additionally, both serum and liver TG levels were markedly decreased in the Con + Ceto group (Figure 7E and F). Histological analysis of liver and mesenteric fat further confirmed that lipid accumulation was lower in the Con + Ceto group than in the Con group (Figure 7I, K, L, and M). To verify whether *C. somerae* intervention promoted mitochondrial fatty acid *β*-oxidation, the protein expression levels of CPT1A and ACAA2 in the liver were significantly elevated in the Con + Ceto group (*P* < 0.05; Figure 7G, H, and J).

### Microbial metabolite 4-trimethylammoniobutanoate (TMAB) from *C. somerae* Boosts L-Carnitine synthesis in the host-gut interactions

Based on the above findings, the heterogeneity of fat deposition was regulated by the gut microbiota through the promotion of mitochondrial fatty acid *β*-oxidation and L-Carnitine was the distinguishing metabolite. To determine whether L-Carnitine originates from the host or the gut microbiota, we conducted integrated analyses of the host transcriptome, metagenome and the whole-genome analysis of *C. somerae*.

**Figure 8.**
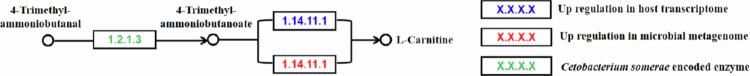
Host and gut microbiota L-Carnitine biosynthesis is promoted by *C. somerae*-produced TMAB. Red indicates upregulation in the Low group of microbial metagenome; blue indicates upregulation in the Low group of host transcriptome; Green indicates *C. somerae* encoded enzyme.

Whole-genome sequencing of *C. somerae* revealed that this strain lacks the full complement of genes required for *de novo* L-Carnitine biosynthesis. However, it encodes [EC:1.2.1.3], an enzyme that catalysers the conversion of 4-trimethylammoniobutanal to TMAB—a key intermediate in the L-Carnitine synthesis pathway (Figure S6A). Targeted metabolomic analysis of the fermentation supernatant confirmed a significant accumulation of TMAB (Figure S6F). To further investigate whether this precursor promotes L-Carnitine production, we analysed the host transcriptome and gut microbiota metagenome. The results showed that both the host and gut microbes encoded [EC:1.14.11.1], a key enzyme catalysing the final step of L-Carnitine biosynthesis, and that its expression was significantly upregulated in the *C. somerae*-treated or Low group (Figure S6B-E and Figure 8). These findings collectively suggest a synergistic mechanism whereby *C. somerae* contributes a critical precursor, while the host and gut microbiota cooperate to complete L-Carnitine biosynthesis.

## Discussion

Excessive lipid deposition is detrimental to host health and is closely associated with metabolic dysregulation, thereby increasing the risk of metabolic disorders such as obesity, type 2 diabetes, non-alcoholic fatty liver disease (NAFLD), and cardiovascular disease.^19^^,^^20^ Regulating gut microbial homoeostasis to optimise lipid metabolism in host has been recognised as an effective strategy for maintaining overall health. In this study, we found significant differences in the gut microbiota between Nile tilapia with high and low MFI. Furthermore, integrated microbiomic and metabolomic analyses suggested that *C. somerae* may inhibit fat deposition by regulating fatty acid degradation. Subsequently, *C. somerae* was confirmed to effectively mitigate mesenteric fat deposition in tilapia. Mechanistically, *C. somerae* was found to harbour the gene encoding [EC:1.2.1.3], facilitating the generation of TMAB, which may participate in enhancing L-Carnitine synthesis in both the host and the gut microbiota to modulate lipid metabolism in fish, consequently impacting fat accumulation.

An expanding body of research underscores the pivotal role of gut microbiota in shaping host phenotypes, including growth,^21^oxidative stress,^16^and lipid metabolism.^22^ In this study, we identified marked differences in gut microbiota composition between high MFI and low MFI fish, with liver fatty acid degradation genes and serum L-Carnitine significantly enriched in the low MFI individuals. Our findings are in concordance with prior research across multiple species, including humans,^23^ mice,^14^ chickens,^24^ and pigs,^8^^,^^25^^,^^26^ all of which have reported distinct microbial profiles between obese and lean individuals. Notably, the gut microbiota of obese individuals has been implicated in enhancing dietary energy extraction, thereby promoting fat deposition.^6^ These evidences reinforce the intricate relationship between gut microbiota composition and lipid metabolism.

The crucial role of gut microbiota in fat accumulation has been extensively validated through germ-free or broad-spectrum antibiotic-treated animal models and faecal microbiota transplantation. For example, transplantation of faecal microbiota from obese mice into germ-free mice led to a pronounced increase in lipid deposition.^14^ Similarly, when faecal microbiota from Ningxiang pigs were introduced into broad-spectrum antibiotic-treated DLY pigs, the recipients exhibited a reduction in muscle lipid accumulation,^25^ underscoring the influence of microbiota-mediated metabolic modulation. In our study, gut microbiota from high MFI and low MFI tilapia were transplanted into germ-free zebrafish. The results demonstrated that zebrafish colonised with microbiota from high MFI tilapia exhibited greater fat accumulation in the liver and mesentery, whereas those receiving microbiota from low MFI tilapia displayed reduced fat deposition. Furthermore, zebrafish transplanted with microbiota from high MFI tilapia exhibited significantly lower CPT1A levels compared to those receiving microbiota from low MFI individuals. Given the well-established role of CPT1A in promoting mitochondrial fatty acid *β*-oxidation,^27^ this finding suggests a potential mechanistic link between gut microbiota composition and host lipid metabolism.

The gastrointestinal tract harbours approximately 10^7^ to 10^11^ bacteria per gram of intestinal content.^28^ Given the high microbial density and diversity in the gut, microbial biomarkers could contribute to the regulation of host metabolism through interactions with both the host and the gut microbiota.^10^^,^^15^ Therefore, in order to identify the microbial biomarkers, we performed correlation network analysis, random forest modelling, LEfSe analysis, and co-occurrence network analysis, which revealed that *C. somerae* is a key microbial component distinguishing the core microbiota between high MFI and low MFI fish. Previous studies have reported a high relative abundance of *C. somerae* in over 43 aquaculture cases, often showing a negative association with obesity (Figure S7). Consistently, our findings revealed an inverse correlation between intestinal *C. somerae* abundance and MFI. To further explore the functional role of *C. somerae*, we isolated, cultured, and conducted a mono-colonisation experiment in germ-free zebrafish and conventionally raised tilapia, which confirmed the lipid-lowering effects of *C. somerae*. These findings are consistent with those of Xie et al. (2022)^29^ and Zhou et al. (2022),^30^ who demonstrated that fish-derived *C. somerae* plays a role in regulating hepatic fat storage, with fermentation products of *C. somerae* significantly reducing TG levels in common carp (*Cyprinus carpio*) and Nile tilapia. These findings suggested that the regulatory influence of *C. somerae* on lipid metabolism is likely mediated by the interplay between microbial metabolic activity and host lipid homoeostasis.

The gut microbiota is widely believed to mediate its effects through the production of diverse metabolites. L-Carnitine was identified as the most critical metabolite influencing the observed phenotypic changes. This metabolite is widely recognised for its essential role in facilitating the transport of long-chain fatty acids across the inner mitochondrial membrane, thereby promoting mitochondrial fatty acid *β*-oxidation and ultimately reducing lipid storage.^31^ Notably, dietary L-Carnitine has been shown to reduce muscle fatty acid levels in tilapia.^27^ Thus, the increased level of L-Carnitine seems to explain the lower mesenteric fat deposition due to the increased mitochondrial fatty acid *β*-oxidation. Nonetheless, the enhanced mitochondrial fatty acid *β*-oxidation was not attributed to the direct production of L-Carnitine by *C. somerae*, as it is widely believed that animals possess the capacity for *de novo* L-Carnitine biosynthesis,^32^ whereas bacteria generally lack the complete enzymatic repertoire required for its *de novo* synthesis.^33^ Interestingly, various gut bacteria are capable of synthesising L-Carnitine from a range of substrates through distinct enzymatic pathways.^34^ For instance, *Proteus mirabilis*, have been shown to convert crotonobetaine to L-Carnitine via L-Carnitine dehydratase.^35^ *Bacillus subtilis* utilises acetylcarnitine as a precursor, employing acyl-L-Carnitine esterases to generate L-Carnitine.^36^ Moreover, *Nocardia crassa*, *Achromobacter*, and *Aspergilus* can produce L-Carnitine from TMAB via the action of [EC:1.14.11.1].^37^ These findings suggest that gut microbiota can employ multiple biosynthetic routes and substrates to generate L-Carnitine, potentially contributing to the host’s L-Carnitine pool. Unexpectively, *C. somerae* could not produce L-Carnitine through above enzymatic pathways. Therefore, we hypothesise that the increase in L-Carnitine levels may result from cooperative interactions between the host and the gut microbiota, in which *C. somerae* provides L-Carnitine precursors that promote L-Carnitine biosynthesis by both the host and other microbial members. Our *in vitro* determination of mitochondrial fatty acid *β*-oxidation level using ¹ ⁴C-labelled palmitic acid further comfirmed that the fermentation supernatant of *C. somerae* significantly promoted the *β*-oxidation efficiency of the tilapia primary liver cell. Supporting this view, Yin et al. (2023)^25^ demonstrated that mono-colonisation of *Lactobacillus reuteri* in antibiotic-treated pigs and mice enhanced muscle mitochondrial fatty acid *β*-oxidation by stimulating L-Carnitine biosynthesis through host-microbiota cooperation. While our evidence confirms that *C. somerae* is capable of TMAB production, integrated analyses—encompassing whole-genome sequencing, metagenomics, and qPCR validation—support TMAB’s pivotal role in L-Carnitine biosynthesis and the regulation of fat deposition. This finding, however, remains preliminary, as it relies on indirect inference. Future work should supplemente exogenous TMAB to directly confirm its role in promoting host L-Carnitine synthesis and reducing fat deposition.

## Conclusion

This study uncovers a previously unrecognised host–microbiota cooperative mechanism in which *C. somerae* facilitates L-Carnitine biosynthesis by supplying the metabolic precursor TMAB, thereby enhancing mitochondrial fatty acid *β*-oxidation and reducing lipid accumulation. Future research are needed to investigate the potential involvement of other gut microbes and the necessity of microbial-derived L-Carnitine biosynthesis pathways in the regulation of host lipid metabolism.

## Ethics approval and consent to participate

All experiments were performed in accordance with the Guidelines of the Care and Use of Laboratory Animals in China. This experiment was authorised by the Committee on the Ethics of Animal Experiments of East China Normal University (No. F20201002).

## Acknowledgments

We thank the instruments sharing the platform of the School of Life Sciences, East China Normal University. Thanks are also extended to Suowen He for his assistance in the study determination of mitochondrial fatty acid *β*-oxidation level using ¹ ⁴C-labelled palmitic acid.

## Supplementary Material

Supplementary Material Supplementary_TableCleanVersion.

Supplementary Material Supplementary Figures and Methods.

## Data Availability

All sequencing data and assembled genomes have been deposited in the NCBI Sequence Read Archive (SRA) under the following accession numbers: PRJNA1297322—RNA-seq data https://www.ncbi.nlm.nih.gov/bioproject/PRJNA1297322 PRJNA1296757—16S rRNA gene sequencing data https://www.ncbi.nlm.nih.gov/bioproject/?term=PRJNA1296757 PRJNA1296868—Shotgun metagenomic sequencing data https://www.ncbi.nlm.nih.gov/bioproject/?term=PRJNA1296868 PRJNA1267279—Whole-genome sequencing of *C. somerae* https://www.ncbi.nlm.nih.gov/bioproject/?term=PRJNA1267279 All other relevant data are available in the Supplementary Materials.
