# Supplementary material for: Intestinal microbiota contributes to the heterogeneity of fat deposition by promoting mitochondrial fatty acid β-oxidation
Source: Gut Microbes. 2025 Dec 3;17(1):2593076. doi: 10.1080/19490976.2025.2593076 (PMC12688268; doi:10.1080/19490976.2025.2593076)
Supplement: Supplementary Material — Supplementary Figures and Methods. [file KGMI_A_2593076_SM6431.doc]

**Intestinal microbiota contributes to the heterogeneity of fat deposition by promoting mitochondrial fatty acid β-oxidation**

Lukuan Li, Nannan Zhou, Zhe Wang, Tong Wang, Yuexin Wang, Fang Qiao, Zhen-Yu Du, Mei-Ling Zhang*

*Laboratory of Aquaculture Nutrition and Environmental Health (LANEH), School of*

*Life Sciences, East China Normal University, Shanghai 200241, China*

*Institute of Advanced Agricultural Science and Technology*, *East China Normal University*, *Shanghai 200241*, *China*

*Corresponding author:

Mei-Ling Zhang (Email: mlzhang@bio.ecnu.edu.cn)

**Supplementary Results**


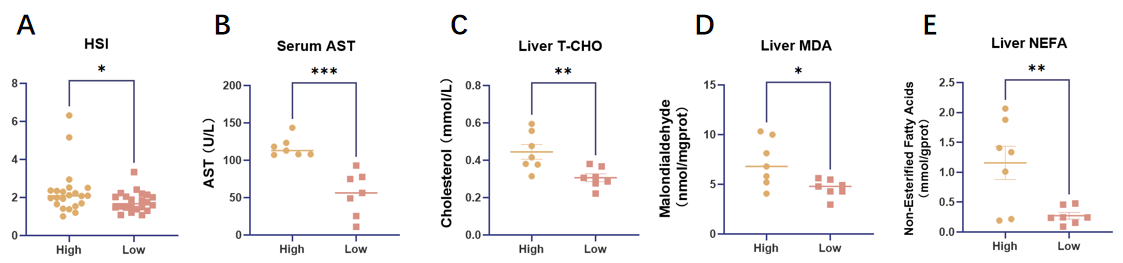


**FIGURES1** Screening of Nile tilapia exhibiting distinct mesenteric fat index (MFI) phenotypes. (A) Statistical comparison of Hepatosomatic Index (HSI) between the High and Low groups. (B) Serum aspartate aminotransferase (AST) content in High and Low groups. (C) Liver total cholesterol (T-CHO) content in High and Low groups. (D) Liver malondialdehyde (MDA) content in High and Low groups. (E) Liver Non-Esterufued fatty aicd (NEFA) content in High and Low groups. Data are presented as mean ± SEM. **P* < 0.05 (Student's t-test). HSI:=100* (Liver weight/Body weight); High group: tilapia with high MFI; Low group: tilapia with low MFI; SEM: standard error of the mean.


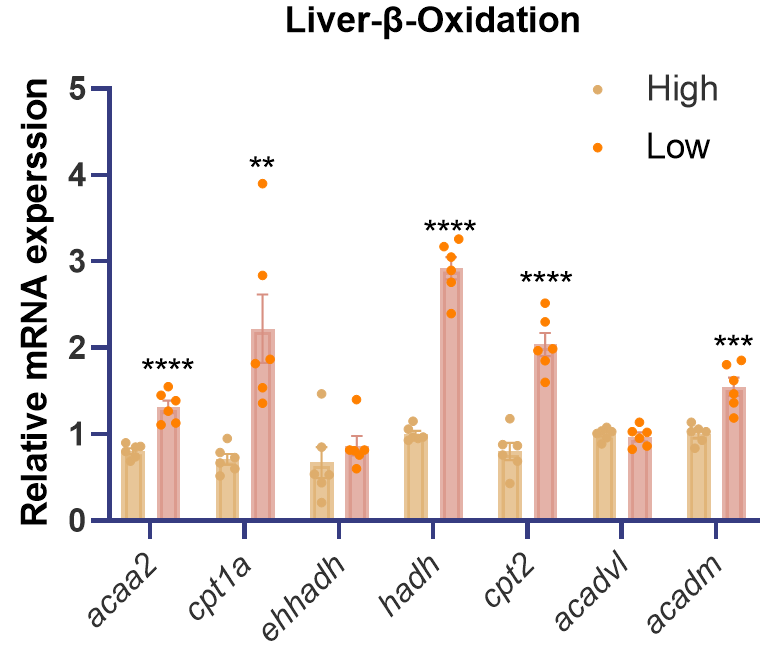


**FIGURES2**. Gene expression of mitochondrial fatty acid β-oxidation in liver tissue between High and Low groups. High group: tilapia with high MFI; Low group: tilapia with low MFI; SEM: standard error of the mean.


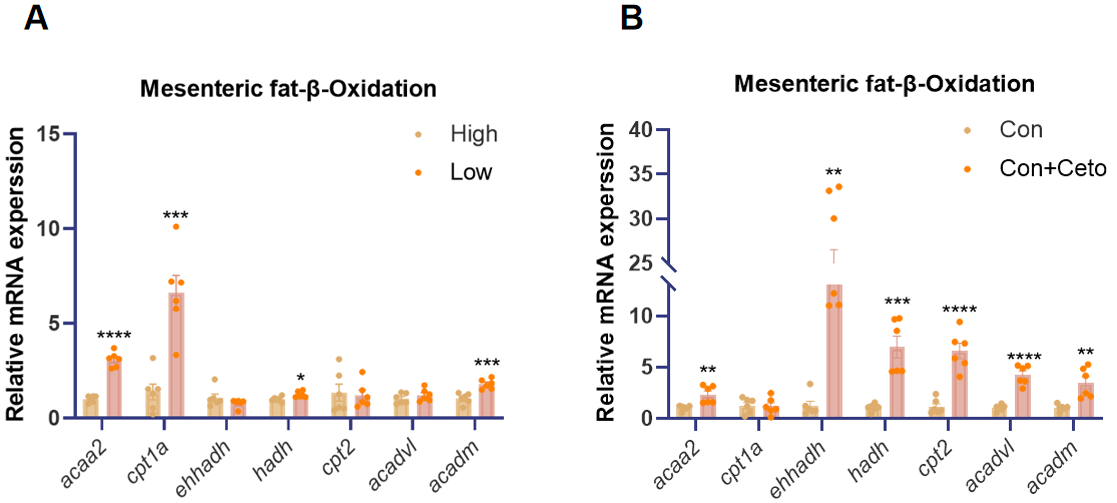


**FIGURES3**. Gene expression of mitochondrial fatty acid β-oxidation in mesenteric fat tissue between High and Low groups. High group: tilapia with high MFI; Low group: tilapia with low MFI; SEM: standard error of the mean.


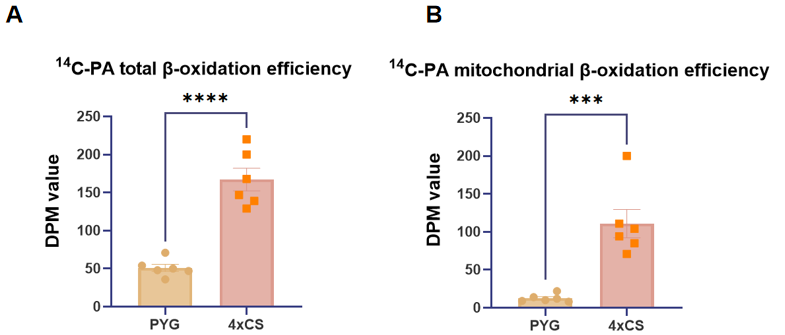


**FIGURES4**. ^14^C-palmitic acid (PA) β-oxidation efficiency in tilapia primary liver cell between PYG and 4×CS groups. (A) ^14^C-palmitic acid total β-oxidation efficiency in tilapia primary liver cell. (B) ^14^C-palmitic acid mitochondrial β-oxidation efficiency in tilapia primary liver cell. PYG: Gifu anaerobic medium; 4×CS is culture supernatant from *C. somerae* at concentrations of 4×10⁸ CFU/mL; SEM: standard error of the mean.


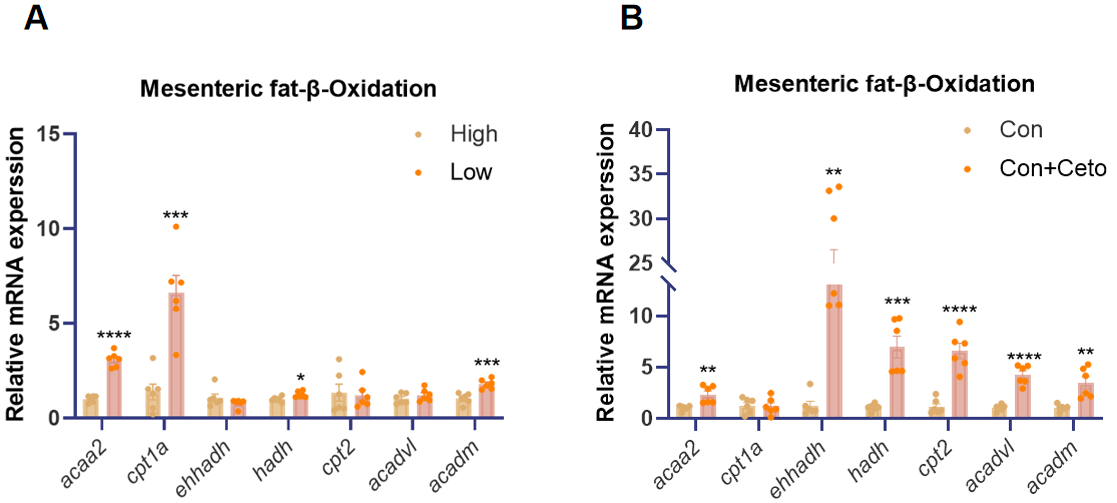


**FIGURES5**. The expression of mitochondrial fatty acid β-oxidation genes between Con and Con+Ceto groups. Con group: tilapia fed with Con diet; Con+Ceto group: tilapia fed with live *C. somerae* diet; SEM: standard error of the mean.


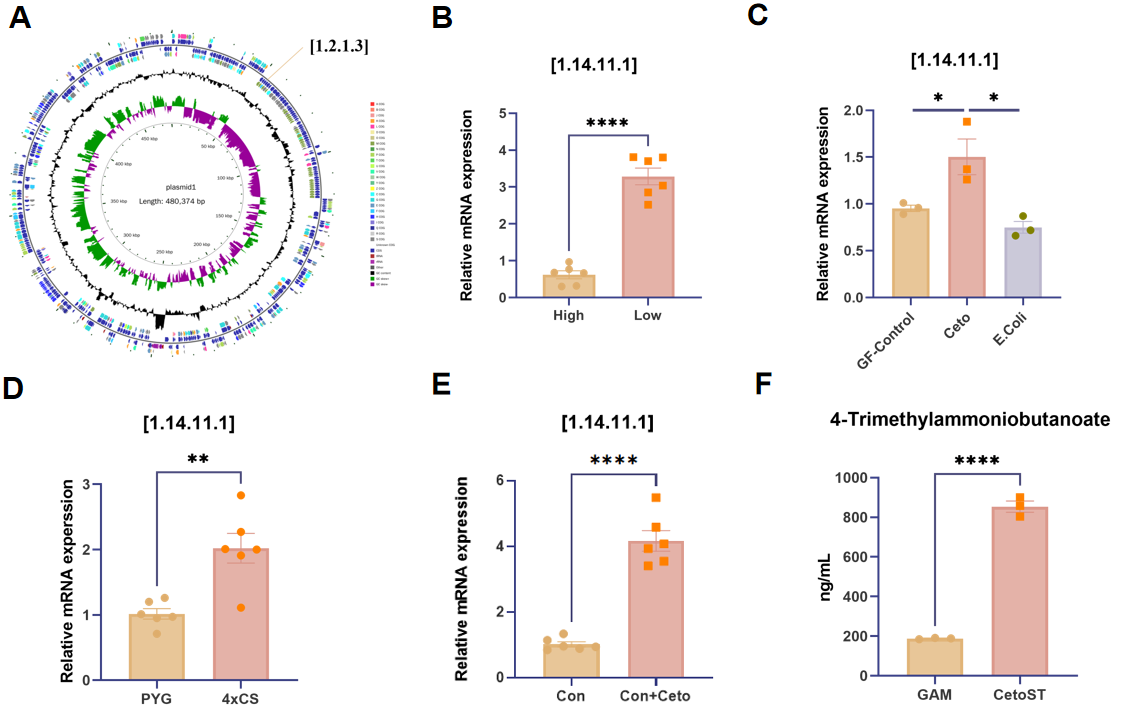


**FIGURES6**. (A) The whole-genome of *C. somerae.* (B) The expression of [1.14.11.1] between High and Low groups. (C) The expression of [1.14.11.1] among GF-Control, Ceto, and *E.Coli* groups. (D) The expression of [1.14.11.1] between PYG and 4xCS groups. (E) The expression of [1.14.11.1] between Con and Con+Ceto groups. (F) The contents of 4-Trimethylammoniobutanoate between GAM and CetoST groups. Data are presented as mean ± SEM. **P* < 0.05 (Student’s t-test). High group: tilapia with a high MFI; Low group: tilapia with a low MFI; GF-Control: Germ-free zebrafish; Ceto: zebrafish colonized with live *C. somerae*; *E. coli*: zebrafish colonized with live *Escherichia coli*; PYG: Gifu anaerobic medium; 4×CS are culture supernatants from *C. somerae* at concentrations of 4×10⁸ CFU/mL, respectively; Con group: tilapia fed with Con diet; Con+Ceto group: tilapia fed with live *C. somerae*diet; GAM: Gifu anaerobic medium; CetoST: *C. somerae* fermentation supernatant; SEM: standard error of the mean.


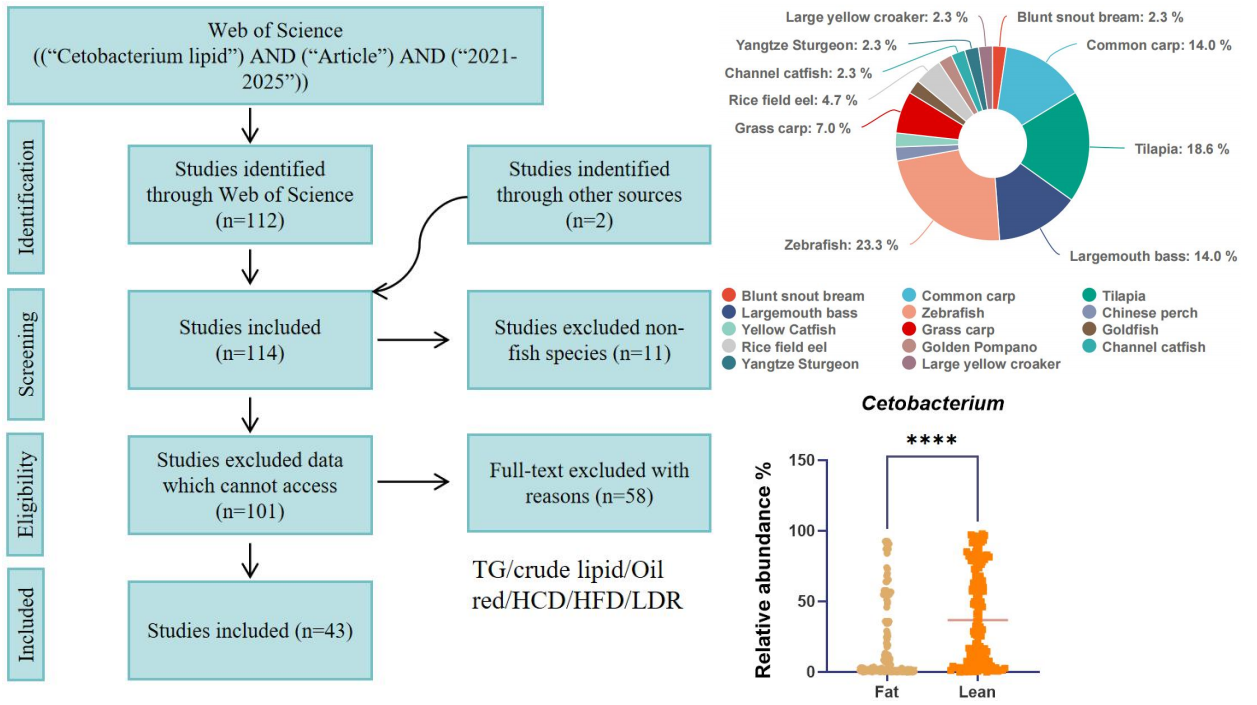


**FIGURES7** Negative correlation between *C. somerae* abundance and obese phenotypes in fish

**Supplementary Methods**

**Production of Germ-Free (GF) Zebrafish**

Natural zebrafish eggs were collected immediately after hatching and transferred into standard zebrafish culture medium. The Germ-free control (GF-Control) embryos were hatched in sterilized gnotobiotic zebrafish medium (GZM), containing 0.06 mg/mL marine salt (Hai Ye, Shanghai, China). The hatched larvae were reared in GZM at 28°C under a constant 14-hour light and 10-hour dark photoperiod, using an external PRX-80 Intelligent Incubator System (Sai Fu, Ningbo, China), with daily media replacement. Specifically, embryos from adult zebrafish were washed three times in sterile water (2 minutes per wash at room temperature) and incubated at 28°C for 5 hours in 50 mL of antibiotic-treated gnotobiotic zebrafish medium (AB-GZM), which contained 0.06 mg/mL marine salt, 100 µg/mL ampicillin, 5 µg/mL kanamycin, and 250 ng/mL amphotericin B. After incubation, the embryos were immersed in a 0.04% polyvinylpyrrolidone (PVP) solution for 40 seconds, washed three times with sterile GZM, then soaked in 0.003% sodium hypochlorite for 10 minutes, followed by three washes in autoclaved sterile GZM. The embryos were reared in 6-well sterile cell culture plates in sterile GZM at a density of approximately 20 embryos per 3 mL of water. All culture conditions were maintained at 28°C with a 14-hour light and 10-hour dark cycle, using the PRX-80 Intelligent Incubator System. To prevent waste accumulation and oxygen depletion, 50% of the GZM in each well was replaced daily with fresh sterile medium. To monitor bacterial contamination, culture media from GF fish were aerobically and anaerobically cultured daily on tryptic soy agar (TSA) and Luria-Bertani (LB) plates at 28℃ and 37°C for at least 48 hours, as described by Rawls et al. (2004).

**Determination of mitochondrial fatty acid β-oxidation level using ¹⁴C-labeled palmitic acid**

After washing the treated primary hepatocytes of tilapia thoroughly with PBS, magnetic beads were added to the cells for homogenization, followed by ultrasonic treatment to further disrupt the cells. The palmitic acid oxidation rate was measured at 28 °C using two culture media, as previously described by (Ning et al., 2016). The first medium permitted both mitochondrial and peroxisomal fatty acid (FA) oxidation, while the second medium allowed only peroxisomal oxidation. After 90 minutes, 700 μL of 10% hydrochloric acid (HClO₄) was added to terminate the reaction. Following centrifugation at 12,000 rpm for 5 minutes, 100 μL of the supernatant was collected and transferred to a scintillation vial preloaded with 2 mL of scintillation medium (Ultima Gold XR, PerkinElmer, USA). Detect the radioactivity in a liquid scintillation counter (Tri-Carb 4910 TR Liquid Scintillation Analyzer, PerkinElmer, USA).

**References**

Rawls JF, Samuel BS, Gordon JI. Gnotobiotic zebrafish reveal evolutionarily conserved responses to the gut microbiota. *Proc Natl Acad Sci USA*. 2004; **101**:4596-601 <https://doi.org/10.1073/pnas.0400706101>

Ning L-J, He A-Y, Li J-M, Lu Dong-Liang, Jiao Jian-Gang, Li Ling-Yu *et al*. Mechanisms and metabolic regulation of pparα activation in nile tilapia (*oreochromis niloticus*). *BBA-MOL CELL BIOL L.* 2016; **1861**:103 6-48. https://doi.org/https://doi.org/10.1016/j.bbalip.2016.06.005
